# Manifesto of a neuroanatomist

**DOI:** 10.3389/fnana.2022.931547

**Published:** 2022-07-18

**Authors:** Javier DeFelipe

**Affiliations:** ^1^Laboratorio Cajal de Circuitos Corticales, Centro de Tecnología Biomédica (UPM), Pozuelo de Alarcón, Madrid, Spain; ^2^Departamento de Neurobiología Funcional y de Sistemas, Instituto Cajal (CSIC), Madrid, Spain

**Keywords:** neuroanatomy, structural design, neural circuits, unexplored brain areas, functional organization

A few years ago I wrote an article entitled “The neuroanatomist's dream, the problems and solutions, and the ultimate aim” (DeFelipe, 2008), to emphasize the critical role neuroanatomy plays in the study of the brain. Although this may appear a rather banal statement, I have realized over the years that this is not so obvious to some colleagues working in other domains of neuroscience (in particular young neuroscientists) and that very little has changed. As I mentioned in the above article, the principal goal in neuroanatomy is to define the detailed structural design of the nervous system. This challenge is one of the first steps toward understanding how neural circuits contribute to the functional organization of the nervous system, both in health and disease. For example, at the electron microscopy level, our mission is to quantitatively analyze key ultrastructural characteristics of axonal and dendritic processes (e.g., density and type of synaptic vesicles, density of mitochondria); synaptic connectivity (e.g., types of synapses, identification of synaptic targets); the relationship between glial processes and synapses, etc. At the system level, neuroscientists strive to establish how brain regions are connected, and how different cell types are distributed across brain regions. Furthermore, knowledge about how brain structures develop and age is essential for understanding brain function from the perspective of lifespan and vulnerability to disease. However, it is frustrating to learn that the importance of neuroanatomy is still not fully appreciated, with little change to the situation I described back in 2008. We often have to face criticisms by reviewers such as “this is an excellent anatomical work, but the *why* is not strong enough”, “the data obtained is interesting but *only* descriptive”, or “the results are great, but if there are no functional studies, the anatomical data do not make much sense.” It seems intuitively obvious that knowing the structure of the brain is essential. It would be naïve to say that the study of the connections of the brain, for example, is merely *important*. The final book of brain anatomy is a long way from being written. There are many unexplored brain areas, particularly in the human, and we are eager to obtain detailed quantitative morphological and neurochemical data on all elements that constitute all regions of the nervous system, as well as their connections, the identification and characterization of neurons and glia, etc. Only when we attain this knowledge may we begin to fully understand the true nature of the human brain. Then we will have a solid foundation for answering with more certainty the fundamental question in neuroscience of how neuronal circuits contribute to the functional organization of the brain, giving rise to cognition and behavior.

## Author contributions

The author confirms being the sole contributor of this work and has approved it for publication.

## Conflict of interest

The author declares that the research was conducted in the absence of any commercial or financial relationships that could be construed as a potential conflict of interest.

## Publisher's note

All claims expressed in this article are solely those of the authors and do not necessarily represent those of their affiliated organizations, or those of the publisher, the editors and the reviewers. Any product that may be evaluated in this article, or claim that may be made by its manufacturer, is not guaranteed or endorsed by the publisher.
